# CB_1_ antagonism increases excitatory synaptogenesis in a cortical spheroid model of fetal brain development

**DOI:** 10.1038/s41598-021-88750-2

**Published:** 2021-04-30

**Authors:** Alexis Papariello, David Taylor, Ken Soderstrom, Karen Litwa

**Affiliations:** 1 Department of Pharmacology and Toxicology, Brody School of Medicine at East Carolina University, Greenville, NC 27834 USA; 2 Department of Anatomy and Cell Biology, Brody School of Medicine at East Carolina University, Greenville, NC 27834 USA

**Keywords:** Cellular neuroscience, Neural circuits, Synaptic development, Induced pluripotent stem cells

## Abstract

The endocannabinoid system (ECS) plays a complex role in the development of neural circuitry during fetal brain development. The cannabinoid receptor type 1 (CB_1_) controls synaptic strength at both excitatory and inhibitory synapses and thus contributes to the balance of excitatory and inhibitory signaling. Imbalances in the ratio of excitatory to inhibitory synapses have been implicated in various neuropsychiatric disorders associated with dysregulated central nervous system development including autism spectrum disorder, epilepsy, and schizophrenia. The role of CB_1_ in human brain development has been difficult to study but advances in induced pluripotent stem cell technology have allowed us to model the fetal brain environment. Cortical spheroids resemble the cortex of the dorsal telencephalon during mid-fetal gestation and possess functional synapses, spontaneous activity, an astrocyte population, and pseudo-laminar organization. We first characterized the ECS using STORM microscopy and observed synaptic localization of components similar to that which is observed in the fetal brain. Next, using the CB_1_-selective antagonist SR141716A, we observed an increase in excitatory, and to a lesser extent, inhibitory synaptogenesis as measured by confocal image analysis. Further, CB_1_ antagonism increased the variability of spontaneous activity within developing neural networks, as measured by microelectrode array. Overall, we have established that cortical spheroids express ECS components and are thus a useful model for exploring endocannabinoid mediation of childhood neuropsychiatric disease.

## Introduction

The endocannabinoid system (ECS) classically regulates synaptic plasticity via inhibitory presynaptic feedback in the adult brain^1^. Constituents of the ECS are also expressed in the human brain during fetal gestation^2,3^, where they direct numerous neurodevelopmental processes, including neural progenitor proliferation^4^, differentiation^5^, neuronal migration^6^, and axonal growth cone directionality^7–9^. While the role of the ECS in the adult brain is well defined, the role of the ECS in the initial establishment of synapses during fetal brain development is not. In the following research, we investigate whether ECS disruption via CB_1_ antagonism impacts synaptogenesis in a cortical spheroid model of fetal brain development.

Synaptogenesis begins when nascent pre- and post-synaptic surfaces contact and adhere to one another^10^. This process requires the coordinated activity of multiple subcellular systems including cell adhesion molecules^11^, scaffold proteins and receptors^11,12^, and cytoskeletal regulators^11,13^. Disruption of these systems which govern synapse selection and maintenance can cause altered excitatory/inhibitory (E/I) synapse balance^10,14,15^. Effective information transfer in the brain relies on homeostatic balance between excitatory and inhibitory synapses, thus, changes to the E/I balance during critical periods of development may negatively impact behavior and cognition^14^. Altered E/I balance is a phenotype which has been implicated in neuropsychiatric ailments that lack clear etiologies including epilepsy^16^, schizophrenia^14,17^ and autism spectrum disorder (ASD)^14^. Thus, while neural circuits are pliant during early development, they are also particularly vulnerable to genetic and environmental disruption.

Many features of neurodevelopmental disorders are difficult to adequately characterize in animal models. This stems from the heterogeneous genetic nature of many neurodevelopmental disorders, the timing of critical periods, and diagnostic criteria that is not easily translated into animal research (such as verbal and nonverbal communication)^18^. Recent advances in the use of induced pluripotent stem cells (IPSC) and the creation of organoid and spheroid model systems promise progress^19,20^. These models, which replicate brain tissue structure better than two-dimensional cell culture^21,22^, provide an avenue for drug testing in a genetically relevant paradigm^23,24^ and allow for the study of human disease processes without complicated in vivo work^20,22^. Using IPSCs from neurotypical control patients, we are able to grow cortical spheroids which have functional synapses, spontaneous activity, an astrocyte population, and pseudo-laminar organization which resembles the dorsal telencephalon of the human fetus at 19–24 weeks post conception^25–27^.

To address how the ECS impacts synapse formation, we focused on the role of endocannabinoid receptor CB_1_. CB_1_ is not only the predominant endocannabinoid receptor in the brain^28^, it is also the most abundant G-protein coupled receptor in the vertebrate central nervous system^29^. Activation of presynaptic CB_1_ by the endogenous, endocannabinoid agonist 2-arachidonoylglycerol (2-AG) elicits activity-dependent, G_i_-linked effects^28,30^ that decrease presynaptic neurotransmitter release and weakens synaptic strength^8,28^. The biosynthetic enzyme for 2-AG, diacylglycerol lipase (DAGLα, the principal CNS isoform), along with the metabolic enzyme monoacylglycerol lipase (MAGL), control the local distribution of 2-AG, which is the principal endocannabinoid during gestation^5,31,32^. In addition to the classic, paracrine signaling of 2-AG during CB_1_-mediated presynaptic feedback inhibition, 2-AG also exhibits distinct, autocrine signaling during development^33,34^. DAGLα colocalization with CB_1_ within the growth cone promotes neuronal polarization and subsequent radial migration by preventing premature synaptogenesis through autocrine, CB_1_ mediated inhibition of presynaptic vesicle exocytosis ^6,34–37^. Once synaptogenesis commences, DAGLα expression in the growth cone decreases while MAGL expression in the nascent presynapse increases^34^. Ultimately, DAGLα localization is redistributed to postsynaptic sites around the somatodendritic axis of mature neurons^34^. This change in enzyme localization is necessary for the switch from autocrine to paracrine 2-AG signaling and facilitates the ability of CB_1_ to regulate synaptic strength. Other important effects mediated by the activation of CB_1_ by 2-AG include neurite retraction^36,38^ and repulsive axonal head movement^39^. Global CB_1_ knockouts in mice, as well as specific interneuron and pyramidal cell CB_1_ knockouts, are viable but show axonal guidance errors and impaired postsynaptic target selection^6,36,37^. Interestingly, pharmacological treatment with both CB_1_ agonists and antagonists creates axon fasciculation errors during development in mice^7,37,40,41^. It is clear from this research that the ECS is necessary for correct axonal targeting and the subsequent establishment of synapses in the developing brain.

Due to the prominent role of the ECS in synapse establishment and maintenance, endogenous or exogenous disruptions to this system during fetal neurodevelopment can impact synaptogenesis and early circuit building. We have developed a model of altered ECS function in cortical spheroids to monitor the effects on synapse formation and the development of synaptic activity in neural circuits. Within our cortical spheroids, we found abundant expression of CB_1_ and ECS-associated enzymes FAAH, MAGL, and DAGLα. Through acute SR141716A (SR) mediated CB_1_ antagonism, we demonstrate that the ECS system regulates the initial establishment of neuronal connections and the resulting synaptic activity. Specifically, SR treatment resulted in a selective and dose-dependent increase in excitatory synapses, and a biphasic response in inhibitory synapses. These complex changes in excitatory and inhibitory synapse formation significantly increased the variability of synaptic activity in developing neural networks. This work establishes cortical spheroids as a powerful model for addressing how endogenous and exogeneous ECS disruption can drive synaptogenesis in neurodevelopmental disorders.

## Methods

### IPSC culture information and techniques

Control WTC-11-ActBmeGFP IPSCs were obtained under MTA from the Coriell Institute. The parental WTC-11 IPSC line was developed by Bruce Conklin of the Gladstone Institute, and was further gene-edited by the Allen Institute for Cell Science using CRISPR/Cas9 to tag endogenous β-actin with monomeric Green Fluorescent Protein (GFP)^42^. Control 7545 19B IPSCs were generated by Dr. Mike McConnell (Lieber Institute for Brain Development) from fibroblasts obtained under MTA from the Coriell Institute. ASD patient IPSCs (UMB#: 5278, 5403, and 797) were obtained under MTA from the NICHD Brain and Tissue Bank for Developmental Disorders. IPSCs were cultured in Matrigel-coated plates and maintained at 37 °C and 5% O_2_ in mTeSR or Essential 8 media. ROCK inhibitor Y-27632 (10 µM) was added to the media during the first 24 h of plating after thawing or splitting cells.

### Cortical spheroid generation and feeding schedule

This protocol is adapted from the Pasca protocol^25^. On day 0, IPSCs were enzymatically lifted off the plate and pelleted for 5 min at 300 rpm. The pellet was disrupted and transferred into 3 wells of a low attachment plate with ESDMEM media (DMEM/F12, 1.5% HEPES, 1% GlutaMAX, 1% NEAA, 10% Knock-Out Serum, and 1% Pen/Strep). ROCK inhibitor Y-27632 (10 µM) and dual SMAD inhibitors SB431542 (10 µM) and Dorsomorphin (10 µM) were added to the media. On day 2, old ESDMEM was replaced with fresh ESDMEM and both SMAD inhibitors were replaced. This protocol continued for the next 3 days. On day 6, dual SMAD inhibitors were substituted with EGF (20 ng/mL) and FGF2 (20 ng/mL) in Neuronal Medium (Neurobasal-A, 2% Gibco B-27 serum substitute without vitamin A, 1% GlutaMax, 1% Pen/strep). Media was replaced every day with fresh FGF2 and EGF for the first 10 days and then every other day for the following 9 days. On day 25, BDNF (20 ng/mL) and NT3 (20 ng/mL) supplementation began and media was changed every other day until day 42. On day 43 all supplements were removed and the Neuronal Media was changed out every 4 days. All cultures underwent regular mycoplasma testing.

### Quantitative real-time polymerase chain reaction

Three independent sets of cortical spheroids from control- and autism derived-iPSCs were used to isolate RNA and synthesize cDNA. For total RNA extraction, spheroids were homogenized in guanidinium-acid-phenol reagent. RNA quality was confirmed by gel electrophoresis. Total RNA (200 ng) was used to synthesize cDNA using an iScript synthesis kit (Bio-Rad). Completed reactions were diluted to a total volume of 200 µL using nuclease-free water and 5 µL was used for each amplification. Each sample set was amplified in triplicate (for an overall total of n = 9 reactions per gene and spheroid type). PCR was done using a kit (SsoAdvanced Universal SYBR Green Supermix, Bio-Rad). Reactions began with a denaturation step for 30 s at 95 C followed by 38 cycles of 95 C × 10 s, 63 C × 30 s. Melt curve analyses were done after reactions were completed to confirm selective amplification. Data were obtained as *C*_*T*_ values using CFX Manager software (Bio-Rad), and the *DDC*_*T*_ method was used to compare expression in control- vs autism-derived spheroids. Primer sequences used can be found in Supplemental Table S1.

### Dosing with SR141716A (SR) for confocal imaging

For confocal imaging, 90-day old cortical spheroids were treated with vehicle, 30 nM, or 300 nM of SR141716A. This CB_1_-selective antagonist/inverse agonist was chosen due to experiments with it in prior work^43,44^. Concentrations employed were determined following dose–response experiments measuring neurite length of IPSC-derived neuronal monolayers (Supplemental Fig. S4). The SR141716A was dissolved in DMSO which also served as the vehicle control. Our vehicle control group was bathed in a final concentration of 0.00001% DMSO, equal to the amount of DMSO added to our 300 nM SR141716A dose groups. Each treatment group consisted of 3–5 midsized (diameter > 1 mm) cortical spheroids. Treatment occurred for 24 h and spheroids were kept in low-attachment plates at 37 °C. This experiment was repeated 3 times with independently grown sets of cortical spheroids. A treatment period of 24 h was chosen to selectively perturb synaptogenesis (rather than migration or differentiation) and prior research from our lab has shown that this is an effective period of treatment for altering synaptogenesis^27^.

### Fixation and cryosection

After 24 h of treatment, spheroids were fixed in 4% paraformaldehyde and cryoprotected in 30% sucrose solution. Spheroids were then placed in OCT embedding medium overnight at 4 °C in a 24 well plate. Spheroids were transferred to a disposable base mold and frozen with dry ice and 2-methyl butane slush. Once frozen, spheroids were sliced 10 µm thick on a cryostat with the objective temperature set at − 7 °C. Mount sections were thawed and transferred on to slides treated with 2% 3-Aminopropyl Triethoxysilane.

### Immunofluorescent staining

A Sequenza rack system was used to stain sectioned cortical spheroids. Slides were blocked in 5% normal goat serum for 30 min. Primary antibodies were diluted in 2% normal goat serum and applied to the slides overnight at 4 °C. Secondary antibodies were diluted in 2% normal goat serum and applied to the slides for 1 h at room temperature in the dark. Slides were removed from the Sequenza rack system and 1.5 mm coverslips were affixed to the slide using Fluorogel with or without DAPI. Slides were placed on a plate warmer for 15 min to ensure coverslip attachment. Cortical spheroids were kept in the dark during the immunofluorescent staining protocol. All antibodies used can be found in Supplemental Table S2. Our CB_1_ antibody was raised to target a 16-amino acid region within the intracellular tail portion of the receptor. The predicted epitope is FRSMFP, corresponding to amino acids 409–414 of human CB_1_. This well-conserved sequence is identical across human, rat, mouse and zebra finch orthologs. Anti-CB_1_ specificity was demonstrated previously by western blot labeling of appropriately sized proteins, expected histological CNS staining patterns and absence of both types of labeling following preincubation with 20 µM of the immunizing peptide^45^.

### Confocal imaging settings and equipment

**Figure 1:**
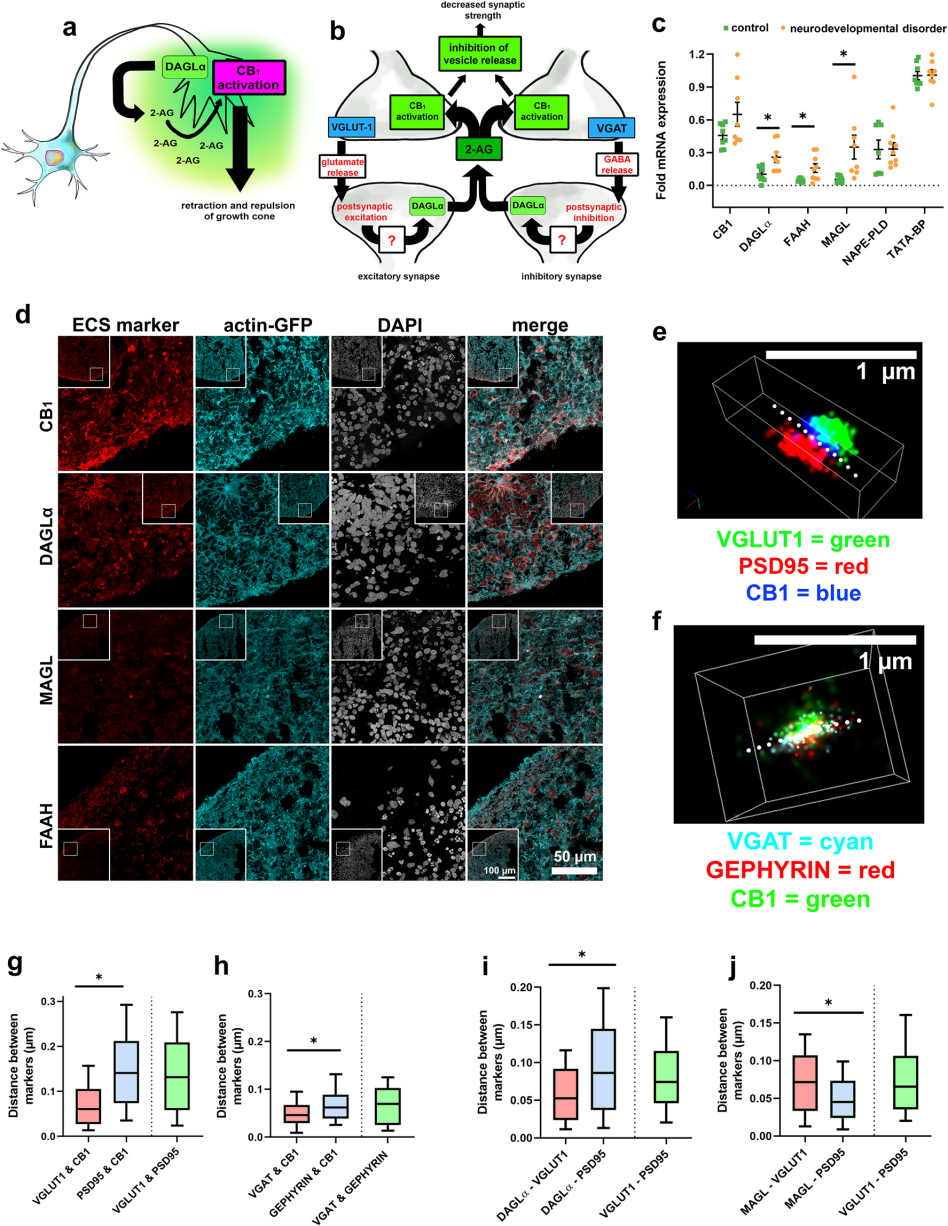
ECS constituents are expressed in 90-day old cortical spheroids. CB_1_, MAGL, DAGLα, and FAAH have increased transcription in cortical spheroids derived from patients with the neurodevelopmental disorder ASD. (**a**) Primary developmental endocannabinoid 2-AG is produced by DAGLα within the growth cone and activates CB_1_ in an autocrine manner during fetal neurodevelopment^33,34^. (**b**) Paracrine signaling mediated by 2-AG at mature synapses regulates both excitatory and inhibitory synaptic plasticity. (**c**) Expression levels of DAGLα, FAAH, and MAGL are elevated in cortical spheroids derived from patients with ASD compared to controls (DAGLα:* p* = 0.007, FAAH:* p* = 0.014, MAGL: *p* = 0.012)**.** Expression levels were assessed by qRT-PCR and are expressed as fold differences from TATA-BP. No significant gene expression differences were observed between control- and ASD-derived samples for CB_1_. Error bars = SEM, compared using student’s t-test. (**d**) Confocal images of 10 µm thick cryosections of 90-day old control cortical spheroids expressing ECS constituents CB_1_, DAGLα, MAGL, and FAAH. All cortical spheroids express endogenous GFP-linked β-actin (actin-GFP). (**e**) Example of an excitatory synapse captured using STORM microscopy. Dashed line represents the synaptic cleft, with CB_1_ (blue) and VGLUT1 (green) on the presynaptic side and PSD95 (red) on the postsynaptic side. (**f**) Example of inhibitory synapse captured via STORM microscopy with CB_1_ (green) located at the presynapse with VGAT (cyan), opposite of gephyrin (red). (**g**) The median distance between CB_1_ and presynaptic marker VGLUT-1 (0.060 µm) is significantly less (*p* < 0.0001, n = 126 synapses) than the distance between CB_1_ and postsynaptic marker PSD-95 (0.141 µm), indicating presynaptic localization. (**h**) The median distance between VGAT and CB_1_ (0.046 µm) was significantly shorter (*p* = 0.014, n = 47 synapses) than the distance between gephyrin and CB_1_ (0.062 µm), indicating presynaptic localization of CB_1_ at inhibitory synapses. (**i**) DAGLα was presynaptic, with a median distance between DAGLα and VGLUT1 (0.052 µm) that was significantly shorter (*p* = 0.006, n = 77 synapses) than the distance between DAGLα and PSD95 (0.086 µm). (**j**) MAGL was postsynaptic, with a significantly shorter (*p* > 0.001, n = 105 synapses) median distance between MAGL and PSD95 (0.045 µm) compared to the distance between MAGL and VGLUT1 (0.072 µm). Distances were compared using the Mann Whitney test, significance was defined as *p* < 0.05. Illustrations in panel a and b were created by Alexis Papariello.

**Figure 2:**
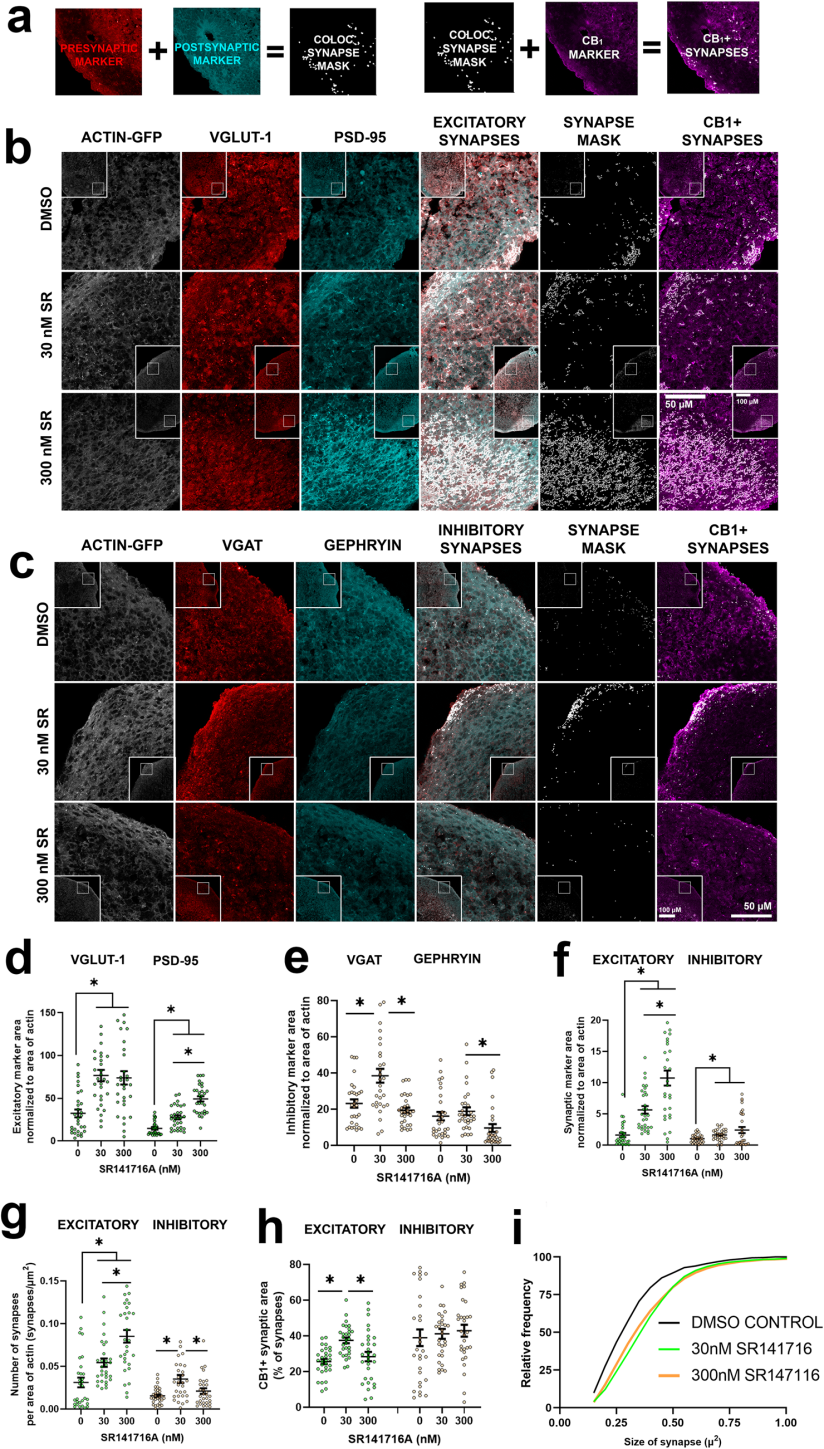
CB_1_ antagonist SR141716A (SR) increases excitatory synaptogenesis in a cortical spheroid model of human brain development. (**a**) Work flow of synapse analysis. (**b**) Representative confocal images of cortical spheroids stained with excitatory presynaptic marker VGLUT1 and excitatory postsynaptic marker PSD95. (**c**) Representative confocal images of cortical spheroids stained with inhibitory presynaptic marker VGAT and inhibitory postsynaptic marker gephyrin. All cortical spheroids endogenously express GFP-linked β-actin (ACTIN-GFP). This was used as the internal control for synaptic area measurements. (**d**) Excitatory presynaptic (VGLUT1) and postsynaptic (PSD95) area after SR treatment. Both high and low doses of SR increased VGLUT1 area compared to vehicle control (0 vs 30 and 0 vs 300: *p* = 0.0001). SR increased PSD95 area in a dose dependent manner (30 vs 300: *p* = 0.0001). (**e**) Box plot of inhibitory presynaptic (VGAT) and postsynaptic (gephyrin) area after SR treatment. The low dose but not the high dose of SR increased VGAT area (0 vs 30: *p* = 0.0035). (**f**) SR increases excitatory synapse area in a dose dependent manner (30 vs 300: *p* =  < 0.0001). SR also increased inhibitory synapse area when compared to control (0 vs 30: *p* = 0.0121, 0 vs 300: *p* = 0.0301) (**g**) SR increases the number of excitatory synapses in a dose dependent manner (30 vs 300: *p* = 0.0030) and increases the number of inhibitory synapses at the low dose when compared to vehicle control (0 vs 30: *p* = 0.0011). (**h**) SR increases the area of CB_1_-positive excitatory synapses at the low dose when compared to vehicle control (0 vs 30: *p* = 0.0001) but has no effect on the ratio of CB_1_-positive inhibitory synapses. (**i**) Cumulative distribution plot of individual excitatory synapse size after treatment with SR. Analyzed by Kolmogorov–Smirnov tests. Data from graphs d-h was analyzed by ordinary one-way ANOVA with multiple comparisons. Data represented as mean ± SEM. n = 90 areas analyzed per dose group. Significance (*) defined as *p* < 0.05.

Imaging took place using ZEN Black Software on a Zeiss Laser Scanning Microscope 700 with a 40 × objective (Plan-Apochromat/1.4 Oil DIC M27). Only cortical spheroids larger than 1 mm were imaged and analyzed. Nine total images per treatment group were taken. Each image consisted of a 4 × 4 tile scan (592.16 × 592.16 µm, 3789 × 3789 pixels) as well as a z-stack. The z-stack was compiled of 5 slices with 1 µm between each slice, making a 4 µm thick stack. Confocal images in Figs. 1 and 2 were pseudocolored from their original, 8-bit, greyscale format. Brightness and contrast have been enhanced equally across controls and experimental groups. Figures were assembled in Adobe Photoshop version 21.2.

### STORM staining and imaging

The preparation of slides for the Nikon STORM microscope follows the same protocol as described above for the confocal with the addition of a 10-min, formaldehyde (4%) and glutaraldehyde (0.1%) post-fix after application of the secondary antibody. Slides were mounted with Vectashield and put onto a plate warmer for 20 min then sealed with nail polish to ensure coverslips were anchored. Stained slides were used within 2 weeks of staining to ensure a robust signal. STORM images were acquired using Nikon NIS-Elements AR software and an Apo TIRF 100 × objective (1.49 NA) on a Nikon Ti-E inverted microscope equipped with N-STORM. Period count was set to 20,000 and laser intensity was set to 100% for all channels.

### Dissociation of cortical spheroids for microelectrode array (MEA) recording

Cytoview 24-well MEA plates with 16 electrodes per well (Axion Biosystems) were prepared with polyethylenimine which was incubated at 37 °C for 1 h. Wells were then washed 4 times with sterile water and allowed to dry out overnight in a sterile hood. Wells were treated with laminin (5 µM) overnight at room temperature after which laminin was replaced with HBSS prior to plating. Cortical spheroids were dissociated onto 24-well MEA plates after 90 days of growth. Between 4 and 6 cortical spheroids were placed into a 1.5 mL tube and washed with ice cold HBSS. Neuronal Isolation Enzyme with papain (Pierce Primary Neuron Isolation Kit, ThermoFisher) was added to the spheroids and incubated at 37 °C for 30 min. Spheroids were dissociated into a single-cell suspension via vigorous pipetting with a 1000 µL micropipette. A cell count was performed with trypan blue. Cells were plated at a density of 250,000 cells per well. The MEA plate was placed in a 37 °C incubator for an hour before MEA media (Neurobasal-A, 2% B27 Plus, 1% GlutaMAX, 1% Pen/Strep) was added to each well. Half of the media was replaced every 3 to 4 days with fresh media.

### MEA recording and SR141716A dosing

MEA recording was performed on day 40 after plating and 48 h after the last feeding. Extracellular recordings were performed in AxIS Naviagtor software using an Axion Maestro Edge set at 37 °C and 5% CO_2_. The recording stream was configured for spontaneous neural bursting activity with network burst detection. Data underwent DC offset filtering and Butterworth band-pass filtering with 0.1 Hz and 5 kHz cutoffs prior to spike detection. A “spike” was defined as a short, extracellular, electrical event with a peak voltage 6 times or greater than the standard deviation of the estimated “noise” signal. A “burst” was defined as 5 or more spikes with no more than 100 ms separating each spike. The MEA plate was placed into the Maestro Edge and activity was allowed to normalize for 5 min prior to a 10-min basal recording. The plate was then removed from the instrument and dosed with vehicle (0.00001% DMSO), 3 nM, 30 nM, or 300 nM of SR141716A in a sterile hood (n = 6 wells per treatment group per independent replicate). The plate was then returned to the instrument and one 10-min recording was taken every hour for 24 h. Recording took place 48 h after the last feeding. Experiment repeated in triplicate with independently grown and plated spheroids.

### Data analysis software and settings

#### Confocal imaging analysis

**Figure 3:**
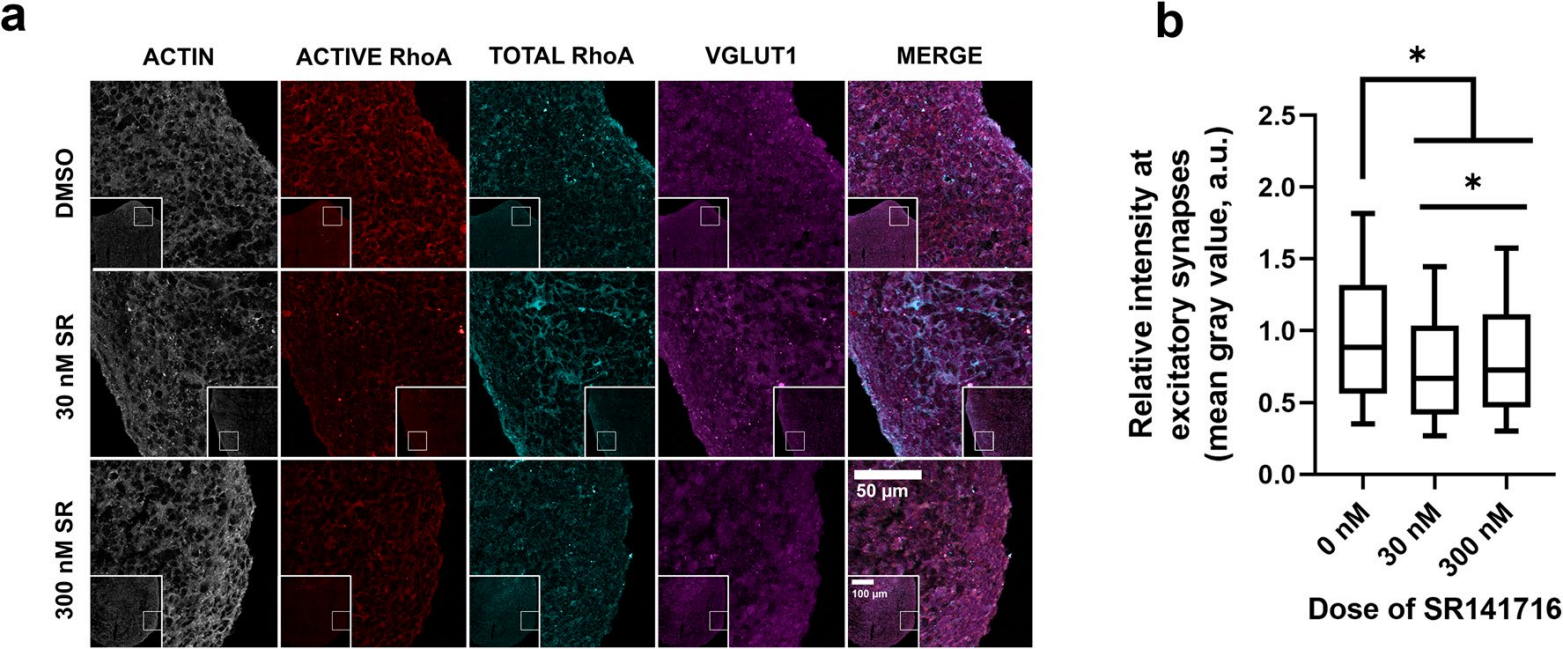
CB_1_ selective antagonism by SR141716A in 90-day old cortical spheroids decreases activity of actin regulator GTPase RhoA (**a**) Representative confocal images of cyrosectioned cortical spheroids stained with Active RhoA, Total RhoA, and excitatory presynaptic marker VGLUT1. Activated RhoA was distinguished from total RhoA by an antibody bound to the GTP form of RhoA (activated). An antibody bound to the GDP form of RhoA distinguished the “total” RhoA. All cortical spheroids express endogenous GFP-linked β-actin (actin-GFP). (**b**) Shown are the normalized ratios of activated RhoA to total RhoA as measured by ratiometric image analysis. Treatment of cortical spheroids with 30 nM and 300 nM SR141716A decreased the relative intensity of gray values at sites colocalized with excitatory presynaptic marker VGLUT1 (0 nM vs 30 nM: *p* > 0.001, 0 nM vs 300 nM: *p* > 0.001). The significant decrease of gray value intensity indicates decreased RhoA activation in the dose groups when compared to the control group. Additionally, an increase in RhoA activity was seen in the 300 nM group (25–75% range: 0.465–1.11 a.u., median: 0.728 a.u. n = 57,577) when compared to the 30 nM group (25–75% range: 0.417–1.03 a.u., median: 0.670, n = 48,899) (30 nM vs 300 nM: *p* > 0.001). Significance determined by Kruskal–Wallis test with significance defined as *p* < 0.05. Control group (0 nM SR) had n = 43,862 VGLUT1-positive areas analyzed. 3 independent sets of 3 cortical spheroids were analyzed per treatment group for a total of 4–6 cortical spheroids analyzed. Normalized mean gray values are reported as relative intensity with arbitrary units (a.u.).

All. czi images were exported from the ZEN Black software as a greyscale .tiff files. Using ImageJ, individual channel z-stacks were consolidated into a max intensity z projection. Each channel had a threshold applied to it. Synapse masks were defined by the coloc_2 plugin using the thresholded presynaptic and postsynaptic images. For Fig. 2, synapse characteristics of area, size, and number were determined using the particle analysis function. For Fig. 3, we used ratiometric image analysis by using the image calculator function in Image J to divide the active RhoA area by the total RhoA area. This area was then overlayed with VGLUT1 area to determine the ratio of activated RhoA at excitatory presynapses. Synapse measurements were taken along the outer edge of the cortical spheroid using 10 × 100 µm diameter circles. Only the outer 100 µm was measured because this is the area of active synaptogenesis in our model at 90 DIV. Within each circle the pre-synaptic, post-synaptic, co-localized pre- and post-synaptic, and CB_1_ staining area was measured. All area values were normalized to the internal GFP-tagged actin. Three images per dose group were analyzed and this process was independently repeated in triplicate. A total of 6–7 cortical spheroids were analyzed per treatment group.

#### MEA analysis

MEA video recording streams (.raw files) were batch processed in AxIS Navigator to detect spontaneous neural spiking activity (.spk). These files were further processed into .csv files by Axion Neural Metrics software. Active electrodes were defined by having 5 or more spikes per minute. The weighted mean firing rate is a measure of spikes per minute within a well and is weighted by the number of active electrodes within that well.

#### STORM analysis

In the NIS-Elements AR program, raw .nd2 files had a constant threshold applied corresponding to the lowest “blink” value in each channel. These files were batch processed into .bin files. Molecular count was performed at this stage. After processing, synapses were located and a z-stack was captured. Subsequent analysis of processed. nd2 files was performed with ImageJ whereby the 3D projection was recreated. A line was drawn across the synaptic cleft and the intensity of CB_1_ as well as pre- and post-synaptic markers was analyzed using the plot profile function of ImageJ.

### Statistical analysis and reporting

Statistical analysis was performed in GraphPad Prism 8. Differences in endocannabinoid gene expression levels in spheroids derived from control vs autism samples were assessed using unpaired two-tailed t-tests. Mann Whitney tests were utilized for STORM distance data. For confocal image analysis, a one-way ANOVA with multiple comparisons was performed across the vehicle control group and both SR dose groups. Stains were normalized to the internal standard of GFP-tagged β-actin. A Kolmogroff-Smirnoff test was performed for the cumulative distribution in Fig. 2. One way ANOVA with multiple comparisons was used for RhoA data. One-way ANOVA with multiple comparisons was utilized for MEA data across time. Dunnett's T3 multiple comparisons test was used for comparison of MEA interquartile ranges. A *p*-value of less than 0.05 was considered statistically significant. All values are reported as mean ± SEM.

## Results

### Cortical spheroids derived from human IPSCs express CB_1_, DAGLα, MAGL, and FAAH

The ECS plays a dual role in the developing brain by modulating both growth cone directionality^7,37,39^ (Fig. 1a) and presynaptic feedback inhibition at mature synapses^28,46^ (Fig. 1b). Disruptions to this system during critical periods of development may have lasting impacts on neural circuitry building. We first examined ECS gene expression levels in cortical spheroids derived from control iPSCs and iPSCs from 3 children with the neurodevelopmental disorder ASD (Fig. 1c). Using quantitative RT-PCR, we found that DAGLα and MAGL, the principal synthetic and metabolic enzymes for 2-AG respectively, were expressed at significantly higher levels in cortical spheroids derived from autistic patients relative to controls (DAGLα: *p* = 0.007, MAGL: *p* = 0.012) (Fig. 1c). Across 3 independent cortical spheroid experiments, DAGLα expression increased from 0.102 ± 0.02 to 0.258 ± 0.05 -fold mRNA expression relative to TATA-BP. Even more dramatically, MAGL expression increased from 0.056 ± 0.01 to 0.351 ± 0.11 -fold mRNA expression relative to TATA-BP. Additionally, the enzyme responsible for anandamide metabolism, FAAH, had significantly higher expression (*p* = 0.014) in ASD spheroids relative to controls and increased from 0.050 ± 0.01 to 0.159 ± 0.04-fold mRNA expression relative to TATA-BP. Significant expression differences were not observed for CB_1_, DAGLβ or NAPE-PLD. We attempted to amplify CB_2_ receptor cDNA but no amplification was observed, leading us to conclude that this receptor is not expressed at detectable levels in our model. The lack of CB_2_ serves as an internal control for our model system because the use of dual-SMAD inhibition blocks mesodermal and endodermal differentiation and therefore the cortical spheroids do not generate microglia, the cell type which primarily expresses CB_2_ in the brain^47,48^.

Using immunofluorescent staining and imaging, we observed abundant cytosolic, synaptic, and neurite expression of CB_1_ in our 90-day old, control patient-derived cortical spheroids (Fig. 1d). We also observed the 2-AG enzymatic regulators, MAGL and DAGLα, in our 90-day old cortical spheroids, as well as FAAH. Interestingly, in concordance with our qRT-PCR results, we found that the area of DAGLα and MAGL was increased in cortical spheroids derived from 2 out of 3 of our ASD patient lines (Supplemental Fig. S1). DAGLα area significantly increased (*p* > 0.001) from 0.109 ± 0.01% in our control patient cell line to 0.795 ± 0.06% and 0.469 ± 0.05% in cortical spheroids derived from ASD patient 1 and 2, respectively. MAGL area was also significantly increased (*p* > 0.001), from 0.636 ± 0.06% in the control patient cell line to 1.34 ± 0.14% and 1.45 ± 0.05% in cortical spheroids from ASD patient 1 and 2, respectively. In the third patient, DAGLα was significantly increased (mean: 0.216 ± 0.02%, control patient vs ASD patient 3: *p* > 0.001) but MAGL was not significantly different from cortical spheroids derived from the control patient IPSCs (mean: 0.462 ± 0.04%) (Supplemental Fig. S1). These findings confirm the presence of the ECS in our cortical spheroid model and suggest increased expression of 2-AG enzymes MAGL and DAGLα may occur in concordance with previous observations of ECS alterations associated with ASD^49,50^.

We wanted to further analyze whether ECS components exhibit synaptic localization characteristic of fetal autocrine signaling. However, confocal microscopy is limited by resolution and prevents us from determining whether ECS components such as CB_1_ correctly localizes to the presynaptic compartment in our system. To overcome this limitation, we used stochastic optical reconstruction microscopy (STORM) which has a resolution of up to ~ 20 nm in x,y, allowing us to resolve individual synapses which have a synaptic cleft distance of ~ 20 nm^26,51^. Examples of excitatory and inhibitory synapses can be found in Fig. 1e, f, respectively. Using STORM, we analyzed 126 excitatory synapses and confirmed presynaptic localization of CB_1_ in our cortical spheroid model, consistent with previous findings in other models^7,52,53^. The median distance between CB_1_ and presynaptic marker VGLUT-1 was 0.060 µm, which is significantly smaller (*p* < 0.0001) than the median distance between CB_1_ and postsynaptic marker PSD-95 (0.141 µm) (Fig. 1g). This indicates that CB_1_ is closer to the presynaptic marker than the postsynaptic marker and preferentially localizes to the presynapse. The median distance between VGLUT-1 and PSD-95 (0.131 µm) was similar to distance between CB_1_ and PSD-95 and is consistent with our previous STORM measurements of synaptic cleft size^26,27^. Additionally, we investigated CB_1_ localization at inhibitory synapses (n = 47) and observed a significantly shorter (*p* = 0.014) median distance between CB_1_/presynaptic marker VGAT (0.046 µm) compared to CB_1_/postsynaptic marker gephyrin (0.062 µm) (Fig. 1h). Further, we observed presynaptic localization of DAGLα (Fig. 1i) and postsynaptic localization of MAGL (Fig. 1j) at excitatory synapses. The median distance between DAGLα and VGLUT1 (0.052 µm) was significantly shorter (*p* = 0.006, n = 77 synapses) than the distance between DAGLα and PSD95 (0.086 µm). MAGL was postsynaptic, with a significantly shorter (*p* > 0.001, n = 105 synapses) median distance between MAGL and PSD95 (0.045 µm) compared to the distance between MAGL and VGLUT1 (0.072 µm). These localizations are consistent with a developmental autocrine CB_1_ signaling paradigm^34,35^.

Since the number of CB_1_ molecules at inhibitory versus excitatory synapses could impact the effect of pharmacological treatment, we used STORM microscopy to analyze the distribution of CB_1_ receptor count at excitatory and inhibitory synapses. We found that CB_1_ receptors are more abundant at excitatory synapses (530 ± 25 CB_1_ molecules/synapse) than at inhibitory synapses (262 ± 14 CB_1_ molecules/synapse) in the outer, 100 µm of the cortical spheroid (*p* > 0.001, unpaired t-test) (Supplemental Fig. S2). Association of the presynaptic terminal with a postsynaptic process is indicative of synapse formation, and increased postsynaptic area is indicative of synaptic strengthening^54^. Our model recapitulates synaptic scaling at both excitatory and inhibitory synapses as demonstrated by the positive relationship between the molecular count of pre- and postsynaptic markers at a given synapse (Supplemental Fig. S2). We therefore sought to determine whether the number of CB_1_ molecules scaled with increased postsynaptic association. We observed a positive relationship between CB_1_ receptor count and postsynaptic marker count at both excitatory (PSD95) and inhibitory (gephyrin) synapses (Sup Fig. S2), suggesting that CB_1_ receptors exhibit synaptic scaling.

Thus, we have determined that human cortical spheroids express ECS machinery, and that CB_1_, the predominant ECS receptor type in the brain, localizes to presynaptic compartments at both excitatory and inhibitory synapses. We also observed DAGLα localization to the presynapse and MAGL localization to the postsynapse in excitatory synapses using STORM microscopy. Our data supports cortical spheroids as a model of the fetal ECS system.

### Treatment with CB_1_ antagonist SR141716A increases the number and total area of excitatory synapses

Having established the expression and presynaptic localization of CB_1_ within our system, we sought to determine how ECS disruption impacts synaptogenesis. In order to selectively perturb CB_1_ during synaptogenesis, we allowed cortical spheroids to develop for 90 days, so as to not disrupt neural differentiation and migration preceding synaptogenesis. At 90 days old, our cortical spheroids model the mid-gestational fetal brain^25^, a critical window of development during which the brain undergoes rapid synaptic proliferation^11^. Disruptions to the spatial and temporal regulation of synaptogenesis during this critical window is thought to drive developmental disorders such as ASD^20,55^. At 90 days of development, we have previously demonstrated that our cortical spheroids exhibit both excitatory and inhibitory synapses^26,27^. Furthermore, these synaptic connections exhibit a high level of plasticity, and are readily altered by acute perturbations to either the intracellular cytoskeleton or extracellular matrix^26,27^. Thus, we have established a window to selectively observe how CB_1_ signaling contributes to the initial formation of synaptic connections and subsequent development of synaptic activity. In order to selectively disrupt the process of synaptogenesis, we acutely treated 90-day old cortical spheroids with selective CB_1_ antagonist SR141716A (SR) for 24 h and observed the resulting effects on excitatory and inhibitory synapses.

Using confocal image analysis (Fig. 2a), we determined the effects of SR treatment on excitatory and inhibitory synaptogenesis by independently measuring pre- and post-synaptic marker area. Cortical spheroids were stained with antibodies against excitatory synaptic markers [vesicular glutamate transporter 1 (VGLUT-1) and postsynaptic density protein 95 (PSD-95)] or inhibitory synaptic markers [vesicular GABA transporter (VGAT) and gephyrin (GEPHRYIN)]. We defined the area of overlap between presynaptic marker (VGLUT-1 or VGAT) and their respective postsynaptic marker (PSD-95 and GEPHRYIN) as a “synapse”. We determined the effect of SR on the number of synapses and size of synapses in the outer 100 µm of the spheroid using this method. Example confocal images used for analysis are given in Fig. 2b, c. Under basal conditions, our cortical spheroids have more excitatory synapses than inhibitory synapses^27^. However, we found that SR treatment impacts both excitatory and inhibitory synapses. SR treatment increased expression of excitatory synapses markers in a dose-dependent fashion, whereas increased inhibitory synapses were only observed at the lower dose of 30 nM SR. To compare the area of synaptic markers across treatment groups, we normalized the area of the synaptic marker to the endogenous β-actin-GFP expression in our cortical spheroids.

The area of excitatory presynaptic marker VGLUT-1 significantly increased from 32.4 ± 4.6% in the vehicle control to 76.7 ± 6.7% and 74.0 ± 7.8% in the 30 nM and 300 nM SR dose groups, respectively (0 vs 30: *p* < 0.001, 0 vs 300: *p* = 0.001) (Fig. 2d). The area of the excitatory postsynaptic scaffold PSD-95 also significantly increased from 14.9 ± 1.4% in the vehicle control to 27.8 ± 2.4% and 49.5 ± 3.0% in the 30 nM and 300 nM SR dose groups, respectively (0 vs 30: *p* < 0.001, 0 vs 300: *p* < 0.001) (Fig. 2d). Additionally, there was a significant, dose dependent relationship between the low and high doses of SR on PSD-95 expression (30 vs 300: *p* < 0.001) (Fig. 2d). Using the colocalization of excitatory presynaptic marker VGLUT-1 and postsynaptic marker PSD-95, we determined that SR treatment significantly increased both the total area (30 vs 300: *p* < 0.001) (Fig. 2f) and number (30 vs 300: *p* = 0.003) (Fig. 2g) of excitatory synapses in a dose dependent manner. Excitatory synapse area significantly increased from 1.6 ± 0.3% in the vehicle control to 5.7 ± 0.6% and 10.7 ± 1.2% in the 30 nM and 300 nM SR dose groups, respectively (0 vs 30: *p* < 0.001, 0 vs 300: *p* < 0.001) (Fig. 2f). Additionally, the number of excitatory synapses per area of actin significantly increased from 0.03 ± 0.005 synapses/µm2 in the vehicle control group to 0.05 ± 0.005 synapses/µm2 and 0.09 ± 0.007 synapses/µm2 in the 30 nM and 300 nM SR dose groups, respectively (0 vs 30: *p* = 0.009, 0 vs 300: *p* < 0.001) (Fig. 2g). The size of individual excitatory synapses trended towards an increase but was found not significant by Kolmogorov–Smirnov test, despite a rightward shift in the cumulative distribution plot (Fig. 2i). Having observed that CB_1_ antagonism increases excitatory synaptogenesis, we also sought to determine whether synaptic CB_1_ distribution was altered in response to SR treatment. We therefore examined CB_1_ localization to excitatory and inhibitory synapses. CB_1_ area as a percent of total excitatory synapse area significantly increased from 25.6 ± 1.5% of total excitatory synapses to 37.5 ± 1.6% percent of total excitatory synapses after application of 30 nM SR (0 vs 30: *p* < 0.001) (Fig. 2h). Surprisingly, the percent of CB_1_-positive excitatory synapses returned to a value similar to the vehicle control after application of 300 nM SR (28.4 ± 2.6% of total excitatory synapses) (0 vs 300: *p* = 0.719, 30 vs 300: *p* = 0.014) (Fig. 2h). Thus, the 30 nM SR treatment captures a window of dynamic ECS alterations at excitatory synapses.

Interestingly, the effect of SR on inhibitory synapses was greatest at the lower, 30 nM dose of SR. The area of presynaptic inhibitory marker VGAT significantly increased from 23.2 ± 2.3% in the vehicle control to 38.5 ± 3.8% in the 30 nM SR dose group (0 vs 30: *p* = 0.003) (Fig. 2e). A decrease in VGAT was observed in the high dose group (19.4 ± 1.5%) when compared to the low dose group (30 vs 300: *p* < 0.001) (Fig. 2e). When compared to the controls, the area of the postsynaptic inhibitory scaffold, gephyrin, was not significantly altered, however, there was a decrease between low and high doses (30 vs 300: *p* = 0.011) (Fig. 2e). There was a significant increase in the area (0 vs 30: *p* = 0.012) (Fig. 2f) and number (0 vs 30: *p* = 0.001) (Fig. 2g) of inhibitory synapses in the low dose SR group compared to the control group. The area of inhibitory synapses increased from 1.02 ± 0.1% in the control group to 1.63 ± 0.2% and 2.41 ± 0.5% in the 30 nM and 300 nM dose groups, respectively (0 vs 30: *p* = 0.012, 0 vs 300: *p* = 0.030) (Fig. 2f). The number of inhibitory synapses per area of actin changed from 0.015 ± 0.002 synapses/µm^2^ in the control group to 0.035 ± 0.005 synapses/µm^2^ and 0.021 ± 0.003 synapses/µm^2^ in the low and high dose groups, respectively (0 vs 30: *p* = 0.001, 30 vs 300: *p* = 0.047) (Fig. 2g). Unlike the results we observed earlier where there was a redistribution of synaptic CB_1_ at excitatory synapses, we did not observe significant differences in CB_1_ localization to inhibitory synapses and the percent of CB_1_-positive inhibitory synapses remained steady at approximately 40% (Fig. 2h).

In order to investigate mechanisms of increased synaptogenesis by CB_1_ antagonism, we used image analysis to measure the ratio of active RhoA to total RhoA (Fig. 3a). CB_1_ activation is associated with rapid growth cone retraction through the GTPase RhoA system^39^; additionally, antagonizing RhoA through ROCK inhibition increases excitatory synapse formation^26^. We therefore sought to determine if CB_1_ antagonism changed RhoA activation through ratiometric image analysis at VGLUT1-positive synapses. Activated RhoA was distinguished from total RhoA by an antibody targeting the GTP-bound form of RhoA compared to an antibody that distinguished total RhoA levels. Treatment of cortical spheroids with 30 nM and 300 nM SR141716A decreased the relative intensity of RhoA activation at excitatory synapses (0 nM vs 30 nM: *p* > 0.001, 0 nM vs 300 nM: *p* > 0.001) (Fig. 3b), consistent with the observed increase in excitatory synapses at these doses.

Thus, using the CB_1_ selective antagonist SR141716A, we successfully manipulated the cortical spheroid system, resulting in increased excitatory synaptogenesis. This increased excitatory synaptogenesis corresponded with increased inhibitory synaptogenesis and CB_1_ expression at excitatory synapses selectively at the lower, 30 nM SR treatment. These results demonstrate the functionality of the ECS in our cortical spheroids and suggest that 30 nM SR treatment could potentially restore excitatory and inhibitory synaptic balance in disrupted systems.

### SR141716A increased variability of synaptic activity as measured by microelectrode array (MEA)

**Figure 4:**
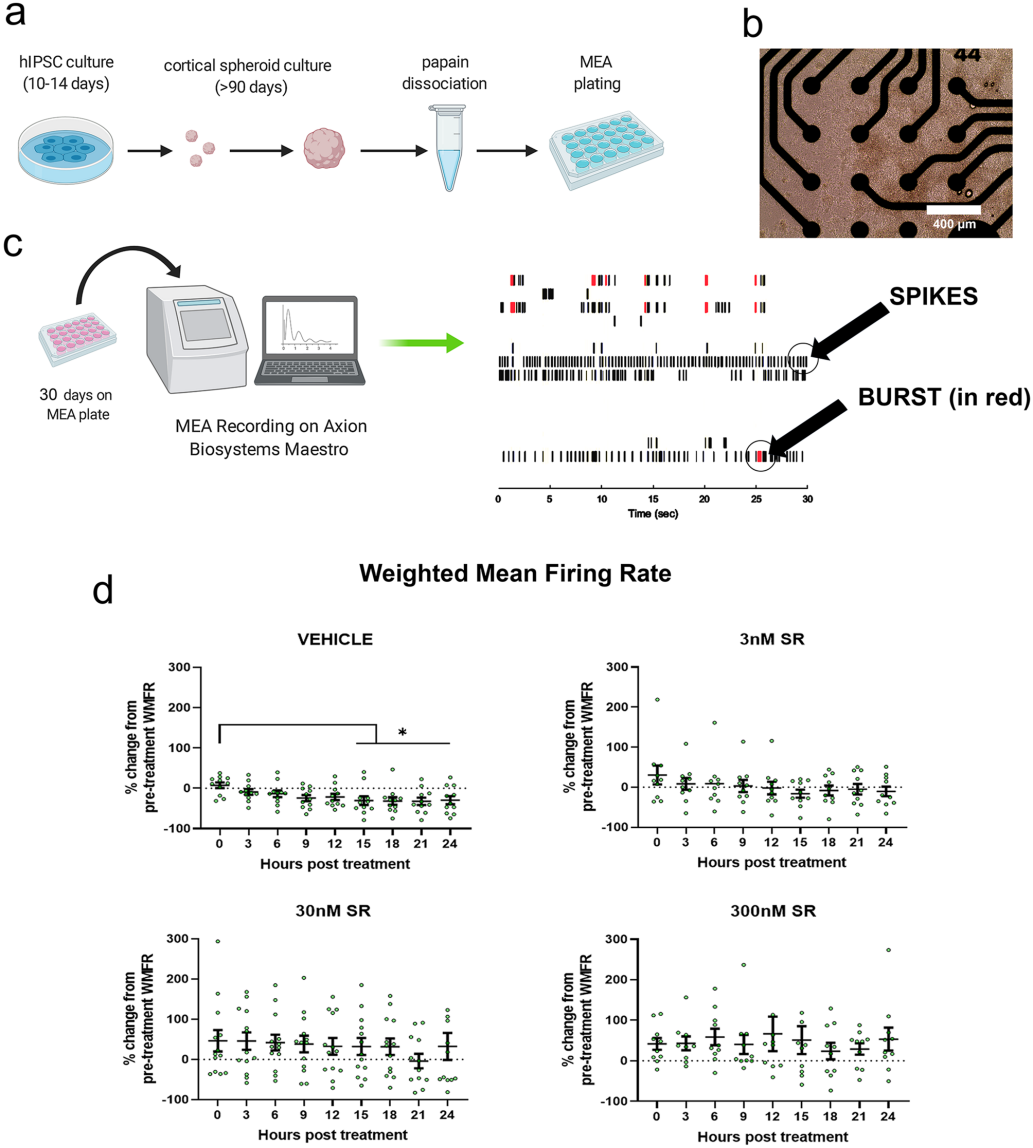

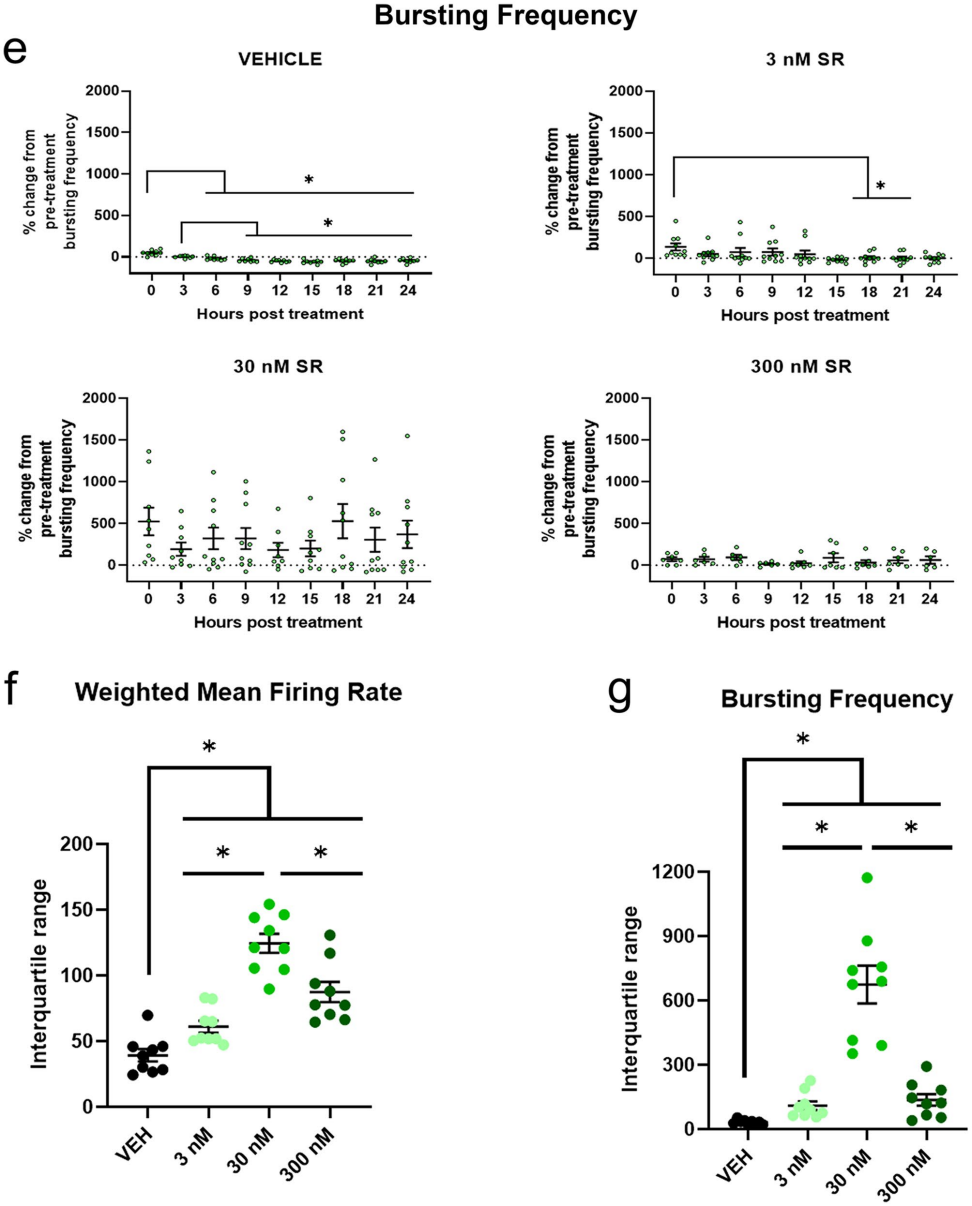
SR141716A did not significantly increase WMFR or bursting frequency but increased variability (**a**) Schematic illustrating the process of cortical spheroid culture, dissociation, and plating. (**b**) Image of dissociated cortical spheroids on top of 16 microelectrodes. Image taken 19 days after dissociation. (**c**) Dissociated spheroids adjust to the MEA plate for 30 days. They are then treated with SR and recorded for 24 h. Raster plots of extracellular activity are analyzed for spiking and bursting activity. (**d**) The weighted mean firing rate (WMFR) of the vehicle control significantly decreased by 50% after 15 h (0 h vs 15 h: *p* = 0.0423, 0 h vs 18 h: *p* = 0.0131, 0 h vs 21 h *p* = 0.0094, 0 h vs 24 h *p* = 0.0099). There was no significant change to WMFR over 24 h of SR treatment. (**e**) Bursting frequency was significantly reduced after 6 h of vehicle treatment (0 h vs 6 h: *p* = 0.0442). (**f**) The average WMFR IQR of the control group over 24 h was 39.3 ± 4.7 compared to 61 ± 4.6 in the 3 nM group, 124 ± 7.3 in the 30 nM group, and 87 ± 7.7 in the 300 nM group. SR treatment significantly increased the WMFR IQR when compared to vehicle treated controls (VEH vs 3 nM: *p* = 0.026, VEH vs 30 nM: *p* < 0.001, VEH vs 300 nM: *p* = 0.001) (**f**). The variability of the 30 nM group was significantly higher than the 3 nM group (3 nM vs 30 nM: *p* < 0.0001) but the variability of the 300 nM group was significantly lower than the 30 nM group (30 nM vs 300 nM: *p* = 0.0166) (**f**) (**g**) Bursting frequency IQR within the control group over 24 h was 32.5 ± 3.5. SR increased the mean IQR to 110 ± 20 in the 3 nM group, 647 ± 88 in the 30 nM group, and 137 ± 27 in the 300 nM group. SR significantly increased bursting frequency variability (VEH vs 3 nM: *p* = 0.0221, VEH vs 30 nM: *p* = 0.0005, VEH vs 300 nM: *p* = 0.0267). Variability was greatest at 30 nM (3 nM vs 30 nM: *p* = 0.0008, 30 nM vs 300 nM: *p* = 0.0009). Effects in panels (**d**) and (**e**) were compared using one-way ANOVA with multiple comparisons. Effects in panels (**f**) and (**g**) were compared using Dunnett's T3 multiple comparisons test. Data represented as mean ± SEM. Significance (*) defined by *p* < 0.05. Schematics in panel (**a**) and (**c**) were created with BioRender (https://biorender.com/).

The effects of cannabinoid modulation on neural activity are complex due to CB_1_ localization at both glutamatergic and GABAergic synapses^30^.To address whether the complex changes in synaptogenesis altered the development of spontaneous activity in neural circuits, we used MEA to measure the extracellular field potential which corresponds to action potential. After 90 days of development, we dissociated cortical spheroids directly onto microelectrodes (Fig. 4a, b). In order to observe consistent activity in our control spheroids, we allow neurons to re-establish connections for an additional month after dissociation, resulting in reproducible activity measurements. We then measure spontaneous neural activity with or without SR treatment. Spontaneous extracellular activity caused by multiple, local action potentials is measured by the electrodes in units called “spikes” (Fig. 4c). Thus, a spike represents an increase in activity across a small area of multiple cells. Multiple spikes of 5 or more in quick succession (< 100 ms between spikes) are defined as “bursts” and represent rapid communication between populations of cells. Synchronous bursting between multiple electrodes is characteristic of mature communication patterns.

We determined that our vehicle decreased the weighted mean firing rate (WMFR) of dissociated cortical spheroids over a period of 24 h. Immediately after dosing, the mean WMFR was 7.64 ± 7.0% above the pre-treatment average, however, after 3 h of treatment the WMFR started to decrease (-9.11 ± 6.95% of pre-treatment) and continued to decrease. The WMFR of the vehicle control group significantly decreased from the initial recording after 15 h of vehicle treatment (0 h vs 15 h: *p* = 0.0423, 0 h vs 18 h: *p* = 0.0131, 0 h vs 21 h *p* = 0.0094, 0 h vs 24 h *p* = 0.0099) (Fig. 4d). We believe that this decrease is attributable to nutrient depletion over time. Interestingly, SR treatment prevented this decrease in WMFR. More strikingly, we observed highly variable activity in response to SR treatment which was not observed in the DMSO vehicle treatment group. WMFR means of the 30 nM and 300 nM group over time were approximately 50% greater than their respective pre-treatment values but there was no significant effect found over time.

The bursting frequency data showed the same trends as the WMFR data. Bursting frequency of the vehicle control group was initially 48.31 ± 10.7% greater than pre-treatment bursting frequency at hour 0, but later decreased to 50% of pre-treatment values after 12 h. Specifically, the vehicle treated wells had significantly decreased bursting frequency continuing after 6 h of treatment (0 h vs 6 h: *p* = 0.0442, 0 h vs 9 h: *p* = 0.0062, 0 h vs 12 h *p* < 0.0001, 0 h vs 15 h: *p* < 0.0001, 0 h vs 18 h: *p* = 0.0010, 0 h vs 21 h: *p* < 0.000, 0 h vs 24 h: *p* = 0.0001) (Fig. 4e). While the low dose of SR showed a significant decrease in bursting frequency after 18 h (0 h vs 18 h: *p* = 0.0440, 0 h vs 21 h: *p* = 0.0308), this decrease took a longer time to manifest when compared to the vehicle treated group (Fig. 4e). Treatment with 30 nM of SR increased bursting frequency by over 200% for every timepoint, but this increase did not vary significantly across time. The high dose of 300 nM SR also increased overall bursting frequency by about 75% but there was no significant effect over time.

While we did not observe significant increases in WMFR and bursting frequency, we did observe a high degree of variability across our SR dose groups. We measured the effects of variability by utilizing the interquartile ranges (IQR) of each timepoint within dose groups and then compared dose groups. The average WMFR IQR of the control group over 24 h was 39.3 ± 4.7 compared to 61 ± 4.6 in the 3 nM group, 124 ± 7.3 in the 30 nM group, and 87 ± 7.7 in the 300 nM group. SR treatment significantly increased the WMFR IQR when compared to vehicle treated controls (VEH vs 3 nM: *p* = 0.026, VEH vs 30 nM: *p* < 0.001, VEH vs 300 nM: *p* = 0.001) (Fig. 4f). The variability of the 30 nM group was significantly higher than the 3 nM group (3 nM vs 30 nM: *p* < 0.0001) but the variability of the 300 nM group was significantly lower than the 30 nM group (30 nM vs 300 nM: *p* = 0.0166) (Fig. 4f). Our bursting frequency results parallel the results of the WMFR, where SR caused significantly more variability compared to the vehicle control (VEH vs 3 nM: *p* = 0.0221, VEH vs 30 nM: *p* = 0.0005, VEH vs 300 nM: *p* = 0.0267) (Fig. 4g). Bursting frequency IQR within the control group over 24 h was 32.5 ± 3.5. SR increased the mean IQR to 110 ± 20 in the 3 nM group, 647 ± 88 in the 30 nM group, and 137 ± 27 in the 300 nM group. Similar to WMFR measurements, we observed a biphasic dose response that displayed significantly more IQR variability in our 30 nM group than in our 3 nM group (3 nM vs 30 nM: *p* = 0.001) and less variability in our 300 nM group when compared to the 30 nM group (30 nM vs 300 nM: *p* = 0.001) (Fig. 4g). Increased variability of synaptic activity, particularly at the 30 nM dose of SR, parallels the complex and differential changes to excitatory and inhibitory synapse formation we observed in our confocal analysis of synaptic area.

## Discussion

Human IPSC-derived cortical spheroids represent a powerful model to explore the effects of genetic and pharmacological manipulation on developing neural circuits which resemble human fetal brain development. The ECS is expressed in human IPSC-derived neurons^38^, mouse IPSC-derived brain organoids^56^, and human IPSC-derived forebrain organoids^57^. Concordantly, we observed ECS expression in both IPSC-derived neurons (Sup. Fig. S3) and cortical spheroids (Fig. 1). Our IPSC-derived neurons displayed a biphasic response to CB_1_ antagonism, where CB_1_ antagonist treatment with 3–300 nM SR abolished WIN-induced neurite length reduction (Supplemental Fig. S4). This indicates that CB_1_ receptors located on neurons which have been differentiated for only 24 h are functional through their response to exogenous cannabinoid modulation. Similarly, recent studies have demonstrated the mutability of the ECS in human-derived brain organoids, specifically showing that 1 µM THC treatment reduces neuronal activity as measured by mean firing rate^58^. Additionally, chronic treatment of dissociated brain organoids with cannabinoid modulators has been shown to produce profound impacts on the process of neuronal differentiation and maturation^57^. With expanding legalization and resultant increased recreational use of cannabis by pregnant women^59^, it is necessary to evaluate the effects of cannabis on fetal neurodevelopment. Importantly, distinctions must be made between acute and chronic maternal use as well as acute and chronic effects of cannabis on the fetal brain. In this research, we sought to characterize the expression and synaptic localization of ECS components in developing neural circuits and to analyze the functional consequences of acute CB_1_ antagonism on synaptic development.

We first wanted to determine if constituents of the ECS were present in our cortical spheroid model and consequently found that CB_1_ mRNA is expressed in our cortical spheroids. Notably, CB_1_ is the predominant cannabinoid receptor in the CNS and one of the most abundant G-protein coupled receptors in the brain^28,29^. CB_2_ mRNA was not detected in the cortical spheroids, however, this differential expression of cannabinoid receptors is in line with our model which does not express microglial cells^25^, the main host of CB_2_ in the brain^47^. We additionally observed differential regulation of DAGLα and MAGL mRNA expression in our cortical spheroids derived from autistic patient IPSCs. Specifically, there was a significant increase in both DAGLα and MAGL mRNA expression. Interestingly, these changes mirror those observed in the mouse model of Fragile X syndrome, where *FMR1* knockout increased striatal DAGLα and MAGL expression^60^. The expression of DAGLα, MAGL, and FAAH dramatically increase in post-natal development, coinciding with a period of synaptic refinement and maturity^61,62^. Thus, increased ECS enzyme levels earlier in development could represent accelerated maturation of neural circuit development, a phenotype which has been observed in neurons derived from autism patients^63^. Pharmaceuticals which alter CB_1_ activation and 2-AG metabolism may be useful treatment options for specific ASD symptoms linked to stress and anxiety, however, the consequences of endocannabinoid manipulation during brain development are unclear and may have unintended results. For example, while the inhibition of DAGL in patients with Fragile X syndrome appears reasonable due to increased DAGL expression in mouse models^60^, reduced 2-AG synthesis is associated with increases in stress^64^, impaired neuroinflammation, and disrupted synaptogenesis^65^. Additionally, MAGL inhibition, while linked to anxiolytic and nociceptive effects^66^, is also associated with impaired learning and memory^67^. Thus, an evaluation of the endocannabinoid system as a primary cause of synaptic dysfunction or as a compensatory mechanism in response to other synaptic changes at the patient level is warranted. We suggest that ECS disruptions may drive the pathophysiology of neurodevelopmental disorders, which would likely disturb the spatial and temporal regulation of homeostatic synapse selection in the developing brain^34^. Additionally, while we believe neurodevelopmental disorders such as ASD are caused by a constellation of deficits that culminate at the synaptic level^14^, we also believe that the disruption of the ECS greatly impacts synaptogenesis and perpetuates synaptic deficits during development.

To determine how antagonism of ECS signaling impacts neural circuit development, we used SR141716A to acutely and selectively disrupt CB_1_ activity during the period of cortical spheroid development coinciding with synaptogenesis. At 30 nM SR, CB_1_ antagonism increased both glutamatergic and GABAergic synaptogenesis. However, at 300 nM, SR continued to increase glutamatergic synaptogenesis but GABAergic synaptogenesis did not significantly differ from the controls. This biphasic effect observed at inhibitory synapses, but not excitatory synapses, is interesting and may be explained by a combination of variables. Firstly, we observe distinct basal expression of CB_1_ at inhibitory and excitatory synapses, with CB_1_ favoring inhibitory synapses (excitatory synapses with CB_1_ = 25%, inhibitory synapses with CB_1_ = 39%, Fig. 2h). This ratio of excitatory to inhibitory CB_1_ expression is comparable to fetal murine models^36,68^, but is notably different from adult CB_1_ expression in the neocortex, where CB_1_ primarily localizes to interneurons^68^. Secondly, we found that CB_1_ receptor count scaled up with both excitatory and inhibitory postsynaptic marker count (Sup Fig. S2), indicating that the number of CB_1_ receptors at the presynapse was a function of synapse size, regardless of type. Our observation of dynamic CB_1_ expression at excitatory synapses during fetal synaptogenesis is consistent with previous literature which describes CB_1_ regulation of glutamatergic neurons from the start of their migration^69,70^ Thirdly, our model system does not have a 1:1 ratio of excitatory to inhibitory synapses in the zone of active synaptogenesis, but rather expresses significantly less inhibitory synapses^27^. Thus, the biphasic effect only observed at inhibitory synapses may due to extraordinarily sensitive CB_1_-positive inhibitory synapses. However, while CB_1_ expressing inhibitory synapses may be more sensitive to the antagonist treatment, we did not observe a decrease in overall activity which would be predicted if CB_1_ antagonism at inhibitory synapses was dominant over CB_1_ antagonism at excitatory synapses. Due to cell-specific and synaptic location-specific effects, it is not fully reliable to characterize ECS regulatory strength based upon the count or density of CB_1_ at excitatory and inhibitory synapses^71^. For example, sparsely expressed CB_1_ on glutamatergic neurons of adult mice plays an outsized role in controlling neural activity in the hippocampus^52^. The disparity between excitatory and inhibitory synapse response to SR141716A in our system may also be explained by the differential expression of glutamatergic and GABAergic neurons across time during development, whereby glutamatergic projection neuron generation and migration occurs prior to interneuron generation^11^.

Complex changes in synaptogenesis mediated by SR141716A were reflected in the variability of neural activity. Under basal culture conditions, spiking and bursting variability decreases across time, mirroring the emergence of synchronized neural networks in the developing brain^21^. Synchronization is a large-scale network process observed in maturing neurons, whereby neuronal spiking activity becomes less variable and larger groups of neurons participate in simultaneous action potentials^72^. In contrast, SR141716A increased the variability of firing activity, indicative of disruption to developing neural networks. Notably, asynchronous activity is observed in neuropsychological disorders such as ASD^73^ and schizophrenia^72^. The disruption of neural synchronicity (i.e. increased variability) was most prominent at 30 nM SR141716A, the dose at which we observed significant increases in both inhibitory and excitatory synapses (Fig. 2f, g). This increase, along with the greater expression of CB_1_-positive excitatory synapses at 30 nM (Fig. 2h) may explain the greater variability of activity. Interestingly, GABA receptor agonists in organoid models decrease synchronicity^21^. Additionally, CCK + interneurons play a role in determining the firing threshold of pyramidal cells in the hippocampus^74^ and may have a similar effect in the cortex. This fact may help to explain why our model experienced greater variability, as CB_1_ antagonism at GABAergic synapses would likely increase GABAergic signaling through the disruption of endocannabinoid-mediated presynaptic inhibition. However, not all inhibitory synapses are the same, and these results are further complicated by the differential impact of CB_1_ at GABAergic perisomatic synapses versus axodendritic synapses^71^. Thus, we report that 30 nM SR141716A results in dynamic ECS alterations that impact synaptogenesis and the resulting neural activity. These complex changes are consistent with the variable and state-dependent response of rat cortical neurons to CB_1_ antagonism^30^. Together, these results demonstrate that ECS signaling critically modulates developing neural circuits by coordinating the proper development and synchronization of excitatory and inhibitory synapses.

The ECS impacts synaptogenesis not only by modulating synaptic strength at mature synapses, but also by influencing axon targeting through autocrine 2-AG release around the growth cone. Prior to synaptogenesis, autocrine 2-AG signaling prevents premature synapse creation by thwarting presynaptic vesicular exocytosis and inducing repulsive, cytoskeletal motility^34,39^. In the presence of SR141716A, this inhibitory process is released and we observed significant increases to excitatory synapse area and count (Fig. 2), indicating an increase in excitatory synaptogenesis. Above average increases in synaptic density during early childhood are a common finding in ASD^20^ and increased glutamatergic synaptic spine density has been observed in post-mortem brains of non-syndromic ASD patients^75^. Excitatory synapse proliferation is physically governed by the cytoskeletal system, which provides the structure of both the pre- and post-synapse. Interestingly, CB_1_ mediated bidirectional modulation of RhoGTPase Rac1 activity has been observed within the growth cone^76^, and CB_1_ agonism induces RhoA kinase (ROCK) dependent growth cone repulsion^36^. The modulation of RhoGTPase activity by CB_1_ provides a direct link between the ECS, the cytoskeletal system, and the regulation of synaptogenesis in neurodevelopmental disorders. Our lab has previously demonstrated that the inhibition of ROCK in cortical spheroids increases excitatory synaptogenesis^26^, mirroring the effects we observed using SR141716A. Thus, CB_1_ antagonism likely prevents CB_1_-mediated, ROCK-dependent repulsion and allows for attractive cues surrounding the growth cone to dominate, ultimately leading to an increase in synaptogenesis.

We have shown that the ECS is present in a cortical spheroid model of fetal brain development and can be antagonized to create a phenotype which displays increased excitatory synaptogenesis and increased variability of neural activity. If the CB_1_ receptor has been likened to a circuit breaker^77^, SR141716A can be likened to a short in the circuit, which interrupts the ability of the breaker to trip and causes the faulty activity to propagate. In this sense, disrupted ECS signaling allows for disrupted synaptic signaling to continue. While synaptic pathologies in neurodevelopmental disorders tend to be propagated by deficits in multiple synaptic regulatory pathways, the ECS plays an outsized role due to the global expression of CB_1_^14^. In this research, we have established that cortical spheroids are an appropriate model for exploring the ECS in the context of fetal brain development and childhood neuropsychiatric disorders. Additionally, we have demonstrated that CB_1_ antagonism produces disruptions to excitatory and inhibitory synaptic balance in cortical spheroids. Our results further confirm the role of the ECS in synaptic pathology and we propose the utilization of CB_1_ as a targetable receptor for therapeutics in neurodevelopmental disorders.

## Supplementary Information

Supplementary Information.
