# Supplementary material for: CB1 antagonism increases excitatory synaptogenesis in a cortical spheroid model of fetal brain development
Source: Sci Rep. 2021 Apr 30;11:9356. doi: 10.1038/s41598-021-88750-2 (PMC8087674; doi:10.1038/s41598-021-88750-2)
Supplement: Supplementary file 1 — Supplementary Information. [file 41598_2021_88750_MOESM1_ESM.pdf]

CB_1 antagonism increases excitatory synaptogenesis in a cortical spheroid model of fetal brain development

Alexis Papariello[1], David Taylor[1], Ken Soderstrom[1*], and Karen Litwa[2*]

[1] Brody School of Medicine at East Carolina University, Department of Pharmacology and Toxicology, Greenville, NC 27834
[2] Brody School of Medicine at East Carolina University, Department of Anatomy and Cell Biology, Greenville, NC 27834
*** Corresponding authors – LITWAK16@ecu.edu and SODERSTROMK@ecu.edu**

Supplemental Material

Supplemental Figure S1

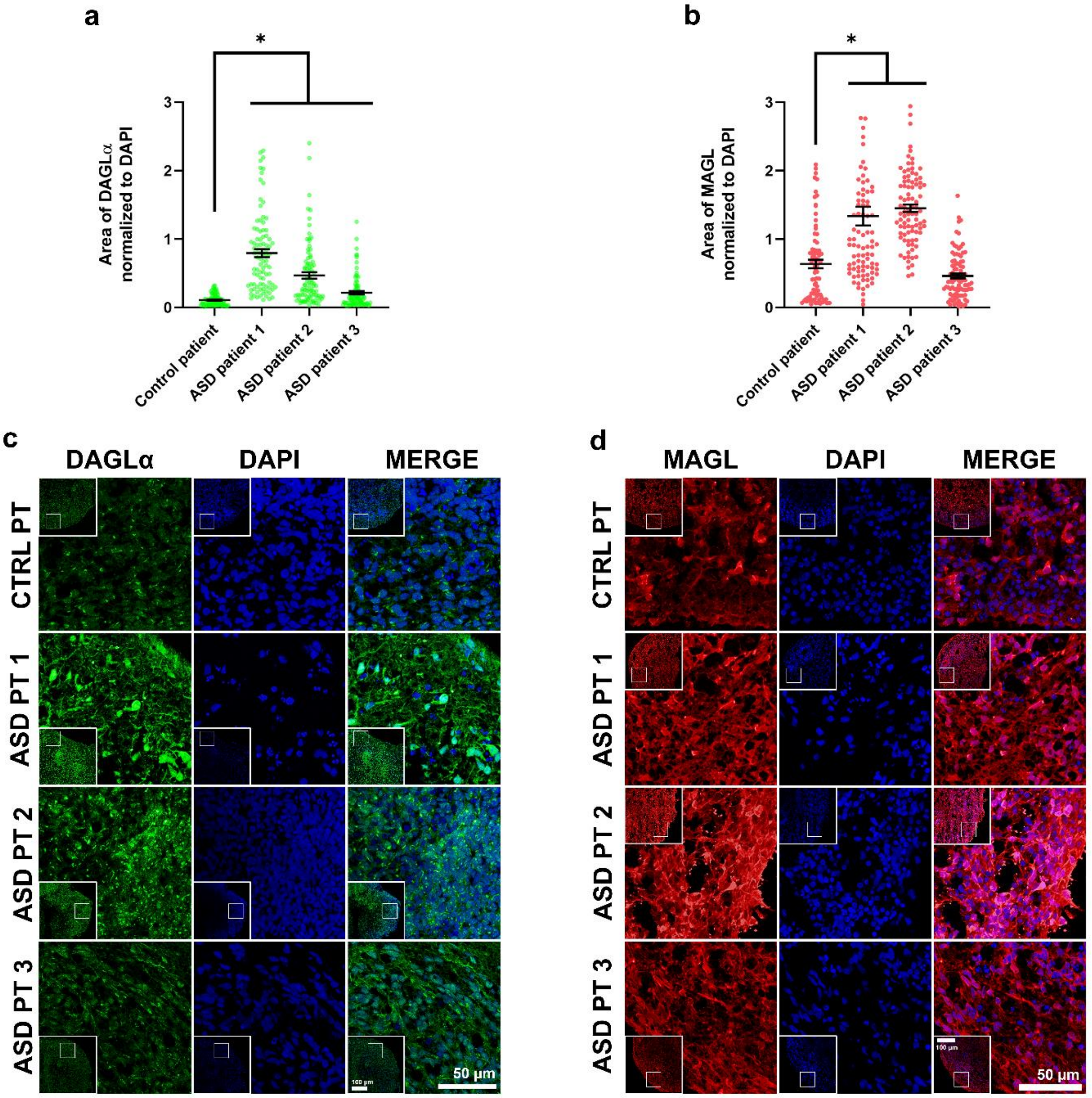

Supplemental Figure 1: DAGLα and MAGL area was increased in cortical spheroids derived from ASD patient lines. (**a**) Using IF image analysis, we found that DAGLα area significantly increased ($p > 0.001$) from 0.109 ± 0.01% in the control patient line to 0.795 ± 0.06% and 0.469 ± 0.05% in the ASD patient lines 1 and 2, respectively. (**b**) MAGL area significantly increased ($p > 0.001$) from 0.636 ± 0.06% in the control patient cell line to 1.34 ± 0.14% and 1.45 ± 0.05% in the ASD patient lines 1 and 2, respectively. In the third patient, DAGLα was significantly increased (mean: 0.216 ± 0.02%, CTRL PT vs ASD PT 3: $p > 0.001$) but MAGL was not significantly different from cortical spheroids derived from the control patient IPSCs (mean: 0.462 ± 0.04%). (**c**) Cyrosections of cortical spheroids derived from control and ASD patient IPSC lines. Spheroids were stained with DAGLα and DAPI. (**d**) Cyrosections of control and ASD patient cortical spheroids stained with MAGL and DAPI. 3 independent replicates of 90 day old cortical spheroids were analyzed for a total of 4-6 cortical spheroids evaluated per cell line. Data represented as mean ± SEM. Significance determined by ANOVA with multiple comparisons against a control. Significance defined as $p < 0.05$.

Supplemental Figure S2

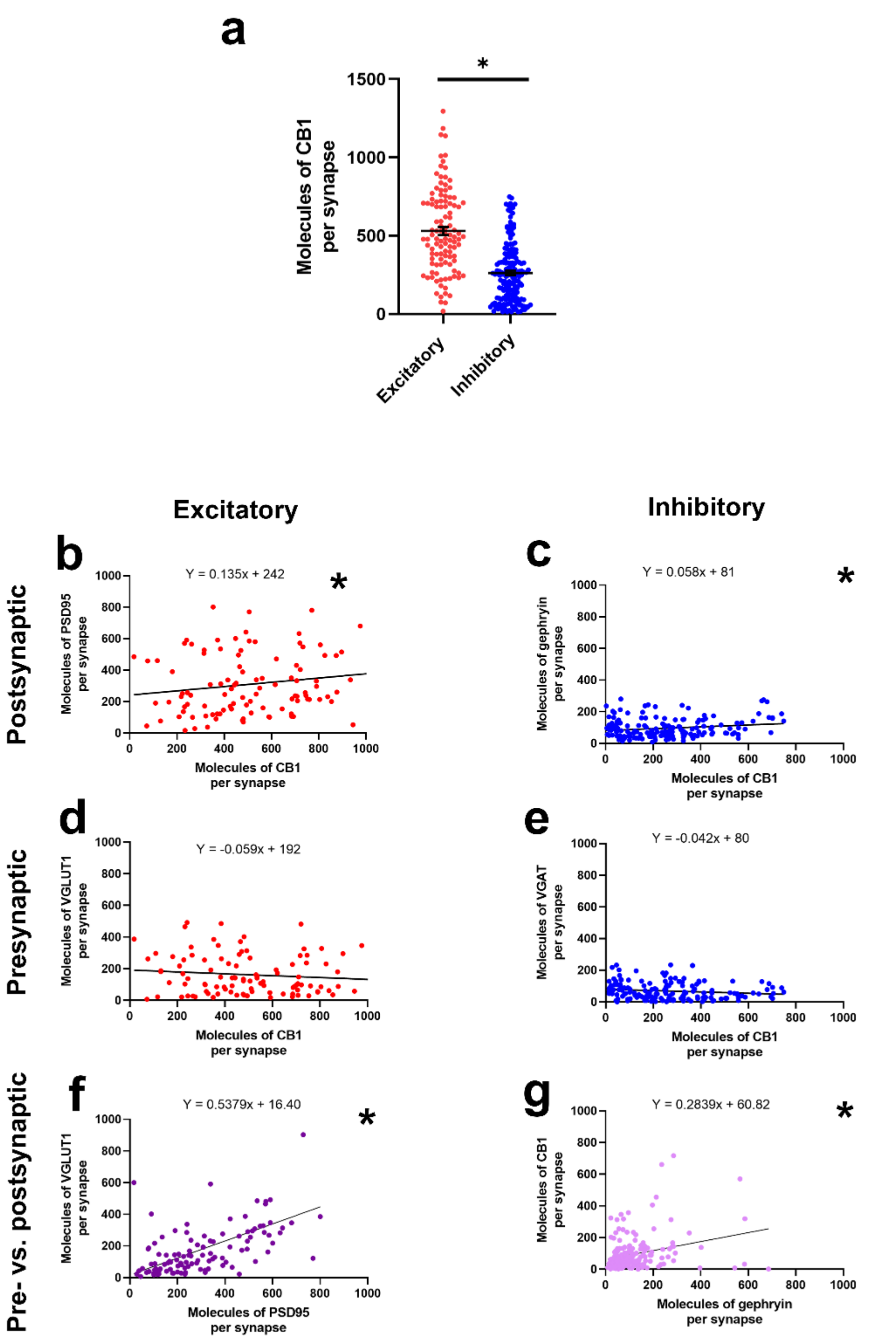

Supplemental Figure 2: **(a)** Using STORM microscopy, we investigated CB_1 molecular count at excitatory and inhibitory synapses and found significantly higher CB_1 molecular count at excitatory synapses (530 ± 25 molecules of CB_1 per synapse) when compared to inhibitory synapse (262 ± 14 molecules of CB_1 per synapse) ($p < 0.001$, Welch's t-test). **(b)** CB_1 receptor count was positively associated with the molecular count of postsynaptic excitatory marker PSD95 ($p = 0.048$). **(c)** CB_1 receptor count was also positively associated with the molecular count of inhibitory synapse marker gephyrin ($p = 0.019$). **(d)** Presynaptic excitatory markerVGLUT1 displayed a nonsignificant relationship with CB_1. **(e)** Presynaptic inhibitory marker VGAT also displayed a nonsignificant relationship with CB_1. **(f and g)** Excitatory synapse markers VGLUT1 and PSD95, as well as inhibitory synapse markers VGAT and gephyrin, displayed positive relationships with one another suggesting synaptic scaling. All measures were taken in the zone of active synaptogenesis (100 µm from the exterior) in 90-day old cortical spheroids. A total of 111 excitatory synapses and 180 inhibitory synapses were measured for the molecular count. For panels b through g, significance was defined by slope deviation from zero via simple linear regression analysis. All values given as mean ± SEM.

Supplemental Figure S3

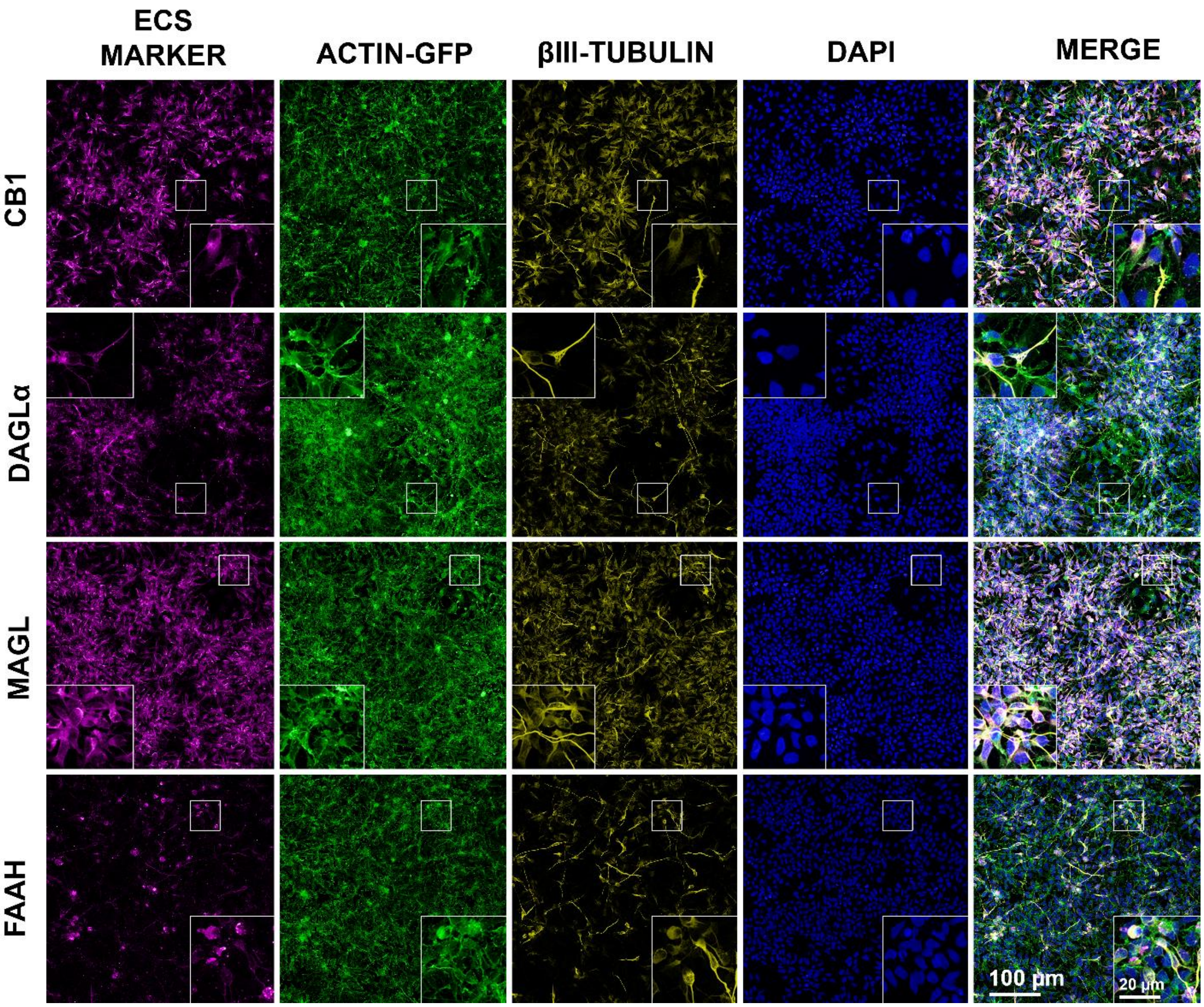

Supplemental Figure 3: Confocal images of 2 week old hIPSC-derived neurons expressing ECS constituents as well as neurite marker βIII-tubulin. Abundant cystolic CB_1, DAGLα, and MAGL was observed in the neurons at this timepoint with some localization of ECS constituents to neurite processes. Neurons were derived from a neurotypical control patient's IPSCs (ActB IPSCs) and have been gene edited via CRISPR-Cas9 technology to endogenously express GFP-tagged β-actin.

Supplemental Figure S4

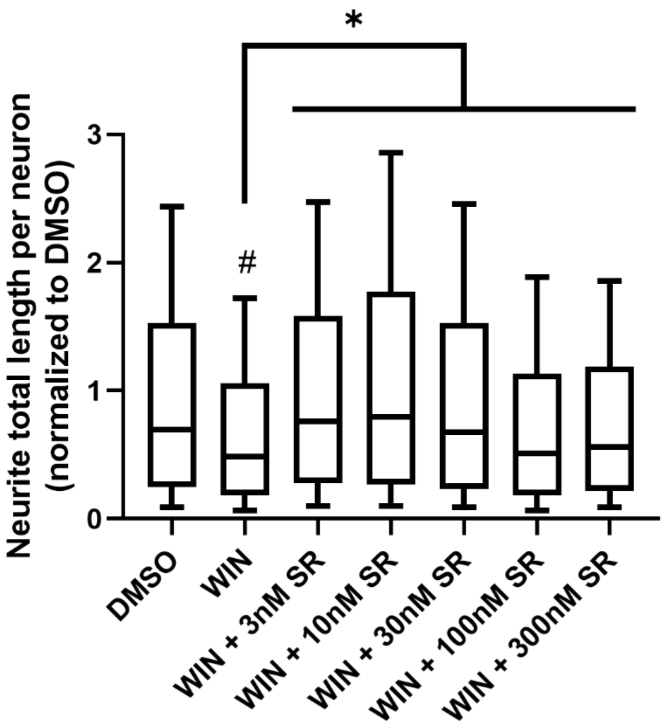

Supplemental Figure 4: We treated 24-hour old, iPSC-derived neurons with vehicle (DMSO), 2µM of CB_1 agonist WIN, or 2µM WIN + CB_1 antagonist/inverse agonist SR for 24 hours. We saw a significant reduction in neurite length after treatment with 2µM WIN compared to vehicle treatment (DMSO median = 0.693, WIN median = 0.484, DMSO vs WIN: $p < 0.001$, as shown by #). This effect of decreased neurite length was reversed with the addition of 3 nM – 30 nM SR (as shown by *, $p < 0.001$). However, we did not see a positive correlation between neurite length and higher doses of SR. The impact of SR treatment on neurite length was greatest at the 10 nM dose (WIN median = 0.484, WIN + 10 nM SR median = 0.796, WIN vs WIN + 10 nM SR: $p < 0.001$). SR at the higher concentrations of 100 nM and 300 nM still showed increased neurite length compared to WIN treated neurons (100 nM SR: $p = 0.011$, 300 nM SR: $p < 0.001$). This implies that SR efficacy is biphasic and distinct in excitatory and inhibitory cells (consistent with data shown in Figure 2). Data represented as box and whisker plots with a range of 10-90%. Significance determined by one-way ANOVA. n = 5000-12000 neurites per dose group analyzed.

Supplementary Table S1

Primers Used for qRT-PCR

Gene Target	Primer Sequence
CB1 Forward	GATGCGAAGGGATTGCCCC
CB1 Reverse	GATGGTGCGGAAGGTGGTAT
CB2 Forward	GTCCTGGGAGAGGACAGAAAAC
CB2 Reverse	GTCTAGAAGGCTTTGGGTTGTG
DAGLα Forward	GAATTCACAAGAGATGCTCCGC
DAGLα Reverse	TCCTCGATGGTGACTCCAGG
DAGLβ Forward	AGGAACAACCAAGAGCCTGC
DAGLβ Reverse	CAGCAGTCACCACCAATCCT
MAGL Forward	GAATGCAAACGCCAGCACAT
MAGL Reverse	TGGGACACAAAGATGAGGGC
FAAH Forward	CTTCACCTACAAGGGCCAGG
FAAH Reverse	TTCCATGGGTTCACGGTCTG
TATA-BP Forward	TTTGCAGTGACCCAGCATCA
TATA-BP Reverse	CCAGCACACTCTTCTCAGCA

Supplementary Table S2

PRIMARY ANTIBODIES

Dilution	Antibody name	Host	Manufacturer	Catalog number
1:1000	VGLUT-1	guinea pig	Synaptic Systems	135304
1:50	PSD-95	mouse	Santa Cruz Biotech	sc-32291
1:500	VGAT	guinea pig	Synaptic Systems	131004
1:500	Gephyrin	mouse	Abcam	ab32206
1:200	CB_1	rabbit	Custom	
1:200	DAGLα	goat	Abcam	ab81984
1:50	MAGL	rabbit	Sigma-Aldrich	ABN1000
1:50	FAAH	rabbit	ThermoFisher	PA5-32183
1:200	GTPase RhoA-GDP (total)	rabbit	Proteintech	10749-AP
1:500	GTPase RhoA-GTP (active)	mouse	ewEast Biosciences	26904

SECONDARY ANTIBODIES

Dilution	Antibody name	Host	Manufacturer	Catalog number
1:500	Atto488	Goat anti-rabbit IgG	Rockland Antibodies and Assays	611-152-1225
1:500	Alexa Fluor 647	Goat anti-guinea pig IgG	ThermoFisher	A-21450
1:500	Alexa Fluor 594	Goat anti-mouse IgG	ThermoFisher	A-11032
1:500	Alexa Fluor 594	Goat anti-rabbit IgG	ThermoFisher	A-11012
1:500	Alexa Fluor 405	Goat anti-mouse IgG	ThermoFisher	A-31553
1:500	Alexa Fluor 594	Donkey anti-goat IgG	ThermoFisher	A-11058
